# Case Report: Multimodality imaging of a primary right ventricular fibroma in an asymptomatic adult

**DOI:** 10.3389/fmed.2026.1878508

**Published:** 2026-06-30

**Authors:** Tian Hu, Wei Li, Hongzhe Tian, Yuhao Liu

**Affiliations:** 1 Department of Medical Imaging, Baoji Central Hospital, Baoji, Shaanxi, China; 2 Department of Imaging, Affiliated Hospital of Yan’an University, Yan’an, Shaanxi, China; 3 Department of Clinical Medicine, General Hospital of Ningxia Medical University, Yinchuan, Ningxia, China

**Keywords:** cardiac tumor, cardiovascular magnetic resonance, case report, right ventricular fibroma, surgical resections

## Abstract

Cardiac fibromas are rare primary benign tumors, predominantly occurring in children; their presentation in asymptomatic adults is uncommon. We report the case of a 23-year-old male with a right ventricular (RV) mass incidentally detected during a routine echocardiographic screening. The mass was characterized using multimodality imaging. Coronary CT angiography (CCTA) delineated its intramural location and identified anatomical relationship with the right coronary artery. Cardiovascular magnetic resonance (CMR) showed uniform T1 and T2 hypointensity alongside intense, homogeneous late gadolinium enhancement, findings characteristic of a dense collagenous matrix. The patient was referred to a specialized center for definitive management, where the tumor was successfully excised. Postoperative histopathology confirmed the diagnosis of a cardiac fibroma. This case demonstrates that integrating CCTA and CMR provides valuable non-invasive characterization differentiating rare cardiac masses and assists in pre-operative planning to mitigate the long-term risk of adverse cardiac events.

## Introduction

Primary cardiac tumors are exceedingly rare, with an autopsy incidence of 0.0017% to 0.03% (1). Among these, cardiac fibroma is a benign connective tissue hamartoma that typically presents in infancy or early childhood (2, 3). Despite their benign histology, giant fibromas pose significant risks, including mechanical obstruction, anatomical compression of the inflow/outflow tracts, and life-threatening ventricular arrhythmias due to the non-conductive nature of the fibrous tissue (4). This case is unique due to the tumor’s exceptional size, its rare location in the RV free wall, and the critical role of advanced imaging in guiding complex surgical resection. Notably, this report illustrates the utility of quantitative T1 mapping CMR findings in characterizing a cardiac fibroma, quantifying the dense collagenous composition of the tumor matrix and providing an objective parametric basis for the non-invasive tissue characterization of intramural masses.

## Case description

This case is reported in accordance with the CARE guidelines (5). A 23-year-old male was admitted to our department on October 30, 2025, following the incidental discovery of a cardiac mass during an occupational health screening. The patient was entirely asymptomatic, denying chest pain, palpitations, syncope, or exertional dyspnea. He had no significant past medical history and no family history of sudden cardiac death or genetic syndromes. Physical examination upon admission was unremarkable, with normal heart sounds and no audible murmurs or rubs.

Upon admission, the patient’s vital signs were stable (BP 133/76 mmHg, HR 72 bpm). Physical examination revealed no jugular venous distention, peripheral edema, or abnormal heart murmurs. Laboratory assessments, including cardiac biomarkers (Troponin T, BNP) and tumor markers (CEA, AFP, CA-125), were all within normal ranges.

Transthoracic echocardiography (TTE) identified a large, well-circumscribed, hypoechoic mass measuring 63 × 50 mm at the right atrioventricular junction. Doppler analysis demonstrated preserved left ventricular function and compression of the tricuspid annulus, with a peak velocity (V*_p_*) of 0.9 m/s (Figure 1).

**Figure 1:**
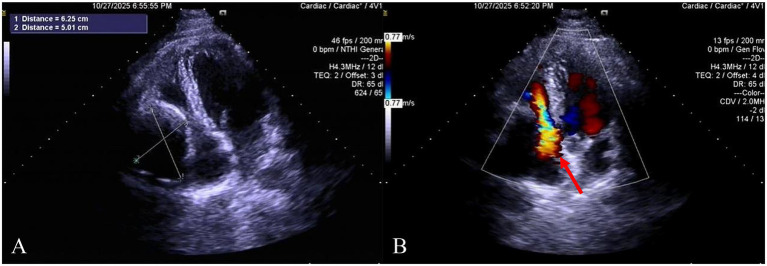
TTE of the cardiac mass. **(A)** 2D TTE showing a well-circumscribed, hypoechoic mass (63 × 50 mm) at the right atrioventricular junction. **(B)** Color Doppler showing tricuspid annulus compression with a peak velocity of 0.9 m/s and preserved left ventricular function.

CCTA conclusively localized the 62 × 47 mm mass within the RV intramural wall. Crucially, the tumor was in close proximity to the right coronary network: the mid-segment of the right coronary artery (RCA) and two acute marginal (AM) branches were adherent to the tumor surface, with the AM branches coursing directly along the tumor’s periphery (Figure 2).

**Figure 2:**
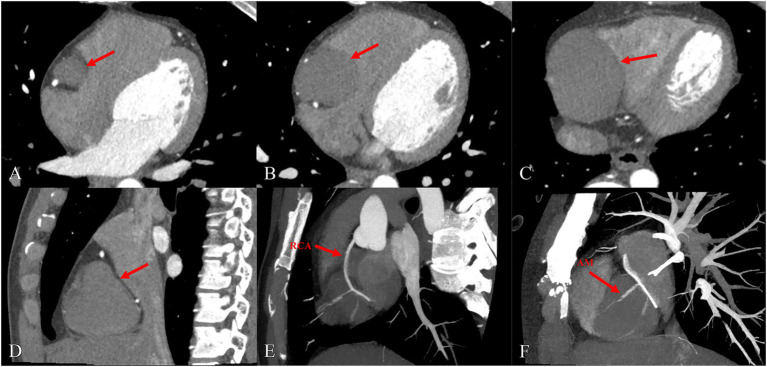
Coronary computed tomography angiography (CCTA) imaging of the right ventricular tumor. **(A–C)** Axial and **(D)** sagittal CCTA images conclusively localize the mass within the right ventricular (RV) intramural wall (red arrows). **(E,F)** Maximum intensity projection (MIP) images demonstrate the close anatomical proximity of the tumor to the right coronary network. The mid-segment of the right coronary artery (RCA) and two acute marginal (AM) branches are adherent to the tumor surface, with the AM branches coursing directly along the periphery of the mass (red arrows).

Cardiac magnetic resonance (CMR) was performed on a 3.0 T scanner (Signa Architect, GE Healthcare). Short-axis and four-chamber cine sequences localized the mass within the right ventricular wall (Figures 3A,B; Supplementary Movies 1, 2). During diastole, the tumor was clearly observed to compress the tricuspid annulus, resulting in anatomical narrowing of the right ventricular inflow tract (Supplementary Movie 1). Subsequent tissue characterization revealed that the lesion exhibited uniform hypointensity on both T1-weighted and T2-weighted sequences (Figures 3C,D). First-pass perfusion imaging demonstrated a complete absence of contrast enhancement within the mass (Figure 3E). Late gadolinium enhancement (LGE) demonstrated intense and homogeneous hyperenhancement (Figure 3F). Quantitative T1 mapping was acquired using a modified Look-Locker inversion recovery (MOLLI) sequence. For parametric assessment, regions of interest (ROIs) were manually placed within the central core of the tumor, yielding a mean native T1 value of approximately 818.5 ms (Figure 3G), which was notably lower than that of the surrounding normal myocardium. Conversely, the post-contrast T1 relaxation time was markedly shortened to approximately 275.3 ms (Figure 3H).

**Figure 3:**
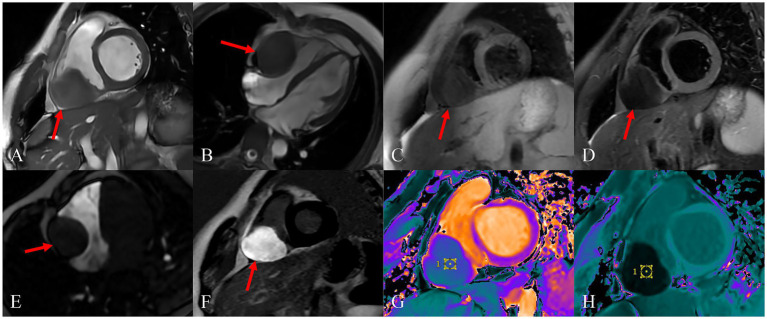
Cardiac magnetic resonance (CMR) imaging of the right ventricular fibroma. **(A)** Short-axis and **(B)** four-chamber cine sequences localizing the mass within the right ventricular wall, demonstrating diastolic compression of the tricuspid annulus and subsequent anatomical narrowing of the right ventricular inflow tract. **(C)** T1-weighted and **(D)** T2-weighted sequences showing uniform hypointensity of the lesion. **(E)** First-pass perfusion imaging demonstrating a complete absence of contrast enhancement within the mass. **(F)** Late gadolinium enhancement (LGE) revealing intense and homogeneous hyperenhancement. **(G)** Native T1 mapping yielding a mean native T1 value of approximately 818.5 ms within the tumor ROI, which is significantly lower than that of the remote normal myocardium. **(H)** Post-contrast T1 mapping demonstrating a markedly shortened T1 relaxation time of approximately 275.3 ms.

Despite the patient’s asymptomatic status, the multidisciplinary team recommended surgical intervention due to the tumor’s significant dimensions and the associated risk of malignant arrhythmias. On November 12, 2025, the patient underwent complete en bloc resection under cardiopulmonary bypass at a tertiary cardiovascular center. Intraoperatively, the mass was found to be firm and white, consistent with fibrous tissue. Meticulous micro-dissection was employed to safely separate the tumor from the right coronary artery (RCA) and the tricuspid apparatus, ensuring the preservation of these structures during resection.

Postoperative gross examination revealed a single, well-circumscribed, grayish-white mass measuring 65 × 64 × 44 mm. The tumor was characterized by a firm consistency, and the cut surface exhibited a distinctive whorled braided appearance. Histopathological analysis confirmed the diagnosis of cardiac fibroma originating from the right ventricle (Figure 4).

**Figure 4:**
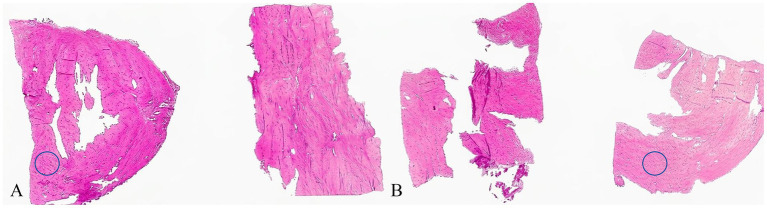
Histopathological examination of the cardiac mass. **(A, B)** H&E stained sections demonstrate a well-circumscribed tumor characterized by a dense, whorled, and braided arrangement of spindle cells within a collagenous stroma, consistent with a cardiac fibroma.

The perioperative period was unremarkable, with no documented surgical complications, including hemorrhage, infection, low cardiac output syndrome, or *de novo* arrhythmias. The patient demonstrated excellent tolerability to the procedure and maintained strict adherence to the postoperative management protocol. The patient’s recovery was uneventful. The patient was discharged on postoperative day 10 in stable condition. At 5-month follow-up, TTE showed no recurrence and complete remodeling of the right ventricle.

## Discussion

This case presents the clinical findings of a 23-year-old asymptomatic male with an incidental discovery of a giant RV fibroma through advanced imaging diagnosis, challenging medical communication, and ultimately, successful surgical resection. Primarily recognized as a benign mesenchymal tumor prevalent in infancy and early childhood, primary cardiac fibromas are extraordinarily rare in healthy young adults (6). Such adult cases are widely postulated to be unrecognized congenital lesions that have undergone decades of indolent growth (7). This report not only enriches the sparse clinical repository of rare adult cardiac tumors and underscores the role of the valuable role of multimodality imaging in non-invasive tissue diagnosis and high-risk surgical planning in the era of precision medicine.

This case illustrates the specific diagnostic utility of various cardiovascular imaging modalities in evaluating right-sided cardiac masses. The differential diagnosis for a RV mass typically encompasses thrombus, infective endocarditis vegetations, and primary benign tumors such as myxoma, lipoma, or hemangioma. Importantly, when relying solely on initial transthoracic echocardiography (TTE) before advanced tissue characterization is available, a pericardial cystic mass lesion must be considered a primary differential diagnosis, given the well-circumscribed, hypoechoic appearance at the right atrioventricular junction (8). Both thrombus and a pericardial cystic mass lesion were excluded by the intense hyperenhancement of the tumor, as thrombi and cysts are avascular and fluid-filled structures that appear nulled on LGE. Myxoma was unlikely given the tumor’s intramural, broad-based attachment and T2 hypointensity, which differ from the mobile, T2-hyperintense, and septal-attached nature of myxomas. Lipoma was ruled out by the T1-hypointense signal, as lipomas are characterized by T1 hyperintensity that suppresses on fat-saturation sequences. The lack of first-pass perfusion further excluded hemangioma, which typically demonstrates robust early enhancement. Finally, the smooth borders, expansile growth, and uniform LGE argued against malignant sarcomas or metastases, which generally present with infiltrative margins, necrosis, and pericardial involvement, thereby supporting a benign etiology.

In the present case, quantitative T1 mapping facilitated the characterization of the tissue properties of the right ventricular fibroma. This parametric imaging modality provided an objective evaluation of the tumor composition, demonstrating a low native T1 value (818.5 ms) and a markedly shortened post-contrast T1 relaxation time. Notably, this native T1 value is substantially lower than the established reference values for normal healthy myocardium at 3.0 T CMR using the MOLLI sequence (septum: 1304 ± 42 ms; mid-left ventricular slice: 1294 ± 37 ms), as benchmarked by Thongsongsang et al. (9). These findings correspond to the dense collagenous stroma and the subsequent retention of the gadolinium-based contrast agent within the matrix. By quantifying these tissue properties, T1 mapping provides a more comprehensive structural characterization of cardiac space-occupying lesions than conventional morphological imaging. Specifically, the combination of a low native T1 value and marked post-contrast T1 shortening provides a robust non-invasive surrogate for the dense, collagen-rich tumor matrix verified by postoperative pathology.

For a massive RV fibroma, surgical resection remains the definitive curative treatment (10). Although the patient was functionally asymptomatic, large fibromas carry an inherent risk of disrupting the cardiac electrophysiological system (11). The interdigitation of dense collagen bundles with normal myocardium uncouples myocytes and generates complex re-entrant pathways. Consequently, malignant ventricular arrhythmias may occur, potentially leading to sudden cardiac death as the initial presentation (12). Thus, current management paradigms suggest that that for large fibromas involving hemodynamic compromise or threatening vital coronary and conduction pathways, complete elective surgical excision should be the primary therapeutic strategy, regardless of symptoms, provided the operative risk is acceptable.

In summary, preoperative CCTA facilitated confirming right coronary trunk patency and defining the tumor’s relationship with the acute marginal branches. This anatomical roadmap enabled complete en bloc resection while preserving the right coronary artery. Following surgery, the patient had an uneventful recovery and has since resumed normal physical activities without residual arrhythmias. These results support early surgical intervention and highlight the value of CCTA and CMR in guiding precision cardiac surgery.

## Limitations

A limitation of this report is the lack of immunohistochemical images, as surgery was performed at an external institution. Furthermore, because the patient transitioned his follow-up to a local hospital closer to his residence, the original postoperative echocardiographic images were unavailable for direct inclusion, although the clinical records confirmed successful right ventricular remodeling and zero recurrence. Lastly, the 5-month follow-up period is relatively short; longer longitudinal surveillance is necessary to definitively rule out late recurrence or delayed complications. However, the diagnosis of fibroma was confirmed by pathological examination, with Masson’s trichrome staining demonstrating extensive collagen proliferation and myofiber infiltration. Given the correlation between preoperative imaging and surgical findings, the diagnostic integrity of this case remains robust.

### Patient perspective

For an asymptomatic 23-year-old, the diagnosis of a massive cardiac tumor with an associated risk of sudden death caused significant initial distress. The discrepancy between his perceived health and the recommendation for invasive open-heart surgery led to initial treatment refusal. However, after reviewing the definitive 3D imaging from CMR and CCTA across multiple institutions, the patient accepted the necessity of surgical intervention. Postoperatively, he experienced a rapid recovery and reported substantial relief. He has since returned to his normal life trajectory, expressing gratitude for the diagnostic capabilities of modern imaging in identifying occult, life-threatening conditions.

## Conclusion

This case highlights the importance of multimodal imaging in diagnosing large, compressive RV fibromas and the success of surgical intervention in mitigating arrhythmia risks. Timely diagnosis and complete resection can lead to an excellent prognosis in young patients.

## Data Availability

The original contributions presented in the study are included in the article/Supplementary material, further inquiries can be directed to the corresponding author.

## Supplementary material

SUPPLEMENTARY MOVIE 1 Cardiovascular magnetic resonance (CMR) four-chamber cine loop demonstrating diastolic compression of the tricuspid annulus and anatomical narrowing of the right ventricular inflow tract.

SUPPLEMENTARY MOVIE 2 Cardiovascular magnetic resonance (CMR) short-axis cine loop delineating the precise tumor morphology and its spatial relationship to the right ventricular intramural wall.
